# The Effect of a Simple Perioperative Video on Maternal Anxiety and Satisfaction Before Regional Anesthesia in a Caribbean Setting: A Randomized Controlled Trial

**DOI:** 10.7759/cureus.36482

**Published:** 2023-03-21

**Authors:** Keevan Singh, Hema Heralal

**Affiliations:** 1 Anesthesia and Intensive Care Unit, Department of Clinical and Surgical Sciences, University of the West Indies, San Fernando, TTO; 2 Department of Anesthesia and Intensive Care, Port of Spain General Hospital, Port of Spain, TTO

**Keywords:** cesarean section, maternal anxiety, anesthesia, spinal anesthesia, obstetrics, educational video, regional anesthesia, anxiety

## Abstract

Background: Anxiety before regional anesthesia and surgery is common among women undergoing cesarean section. Although perioperative education has been shown to reduce this level of anxiety, the optimal form and timing of this intervention are not known. The goal of this study was to evaluate the efficacy of an educational anesthetic video on reducing anxiety and improving maternal satisfaction in patients scheduled for elective cesarean section under regional anesthesia.

Methods: Eighty patients scheduled for cesarean section at a tertiary-level obstetric center were randomized to either an interventional group (viewed an educational video on the evening before surgery) or the control group (no educational video). Both groups received a standard preoperative assessment on the morning of surgery. Anxiety was assessed using the Spielberger State-Trait Anxiety Inventory (STAI) and the visual analog scale for anxiety (VAS-A). Maternal satisfaction was assessed using the Maternal Satisfaction Scale Score for Cesarean Section (MSSSCS). Anxiety was assessed at baseline (the evening before surgery) and immediately before surgery. Maternal satisfaction was assessed on the first postoperative day. Patients in the intervention group also had their state anxiety measured immediately after viewing the educational anesthetic video using the VAS-A.

Results: Both groups were equally matched at baseline, and a statistically significant reduction in anxiety measured using VAS-A was seen in the intervention group vs. the control group (6 vs. 4.6, p = 0.018). State-trait anxiety measured using STAI, however, was not significantly lower in the intervention vs. control group (44 vs. 46, p = 0.99). VAS-A immediately after looking at the video was also not significantly different (5 vs. 4, p = 0.323) from the control group. Maternal satisfaction was also higher in the intervention group (113 vs. 104.5, p = 0.015).

Conclusion: The use of a simple educational anesthetic video may be associated with reduced anxiety and improved maternal satisfaction in patients scheduled for elective cesarean section under regional anesthesia.

## Introduction

Preoperative anxiety is generally described as an unpleasant emotional state secondary to several perioperative factors, including surgery and anesthesia [1]. The psychological effect of elevated anxiety can lead to an increased sedative, anesthetic, and analgesic requirement [2]. Additionally, it can lead to an increased incidence of postoperative nausea and vomiting, longer post-anesthesia care unit (PACU) stay, unanticipated hospital admission, increased postoperative pain, increased analgesic requirements, and delayed recovery [3-5].

Patient-reported anesthesia-related anxiety includes concern about pain during the procedure, being paralyzed after surgery, failure to wake up after surgery, being awake during surgery, seeing or hearing during surgery, and respect for privacy [6,7]. Patients with increased anxiety are more likely to refuse a regional technique, which may lead to an increased number of patients requesting general anesthesia with its inherent risks [8,9].

Preoperative education is associated with a reduction in anxiety and many studies have evaluated the various formats and their anxiolytic effects. The most effective method is still unclear and varies with respect to the study population, the timing of preoperative education, and the design and quantity of information provided [10].

The efficacy of preoperative education via an educational anesthetic video on patients’ anxiety levels is variable. However, the majority of studies did show a decrease in anxiety [11-14].

It is also important to consider that many of these studies have been done in developed countries and may not apply to all socioeconomic and cultural settings. This is especially so in regions where patients may have difficulty accessing and interpreting reliable information. Additionally, the high-quality videos used in many of the studies may not be reproducible in many regions.

We thus sought to create a low-cost educational video and evaluate its effect on maternal anxiety and satisfaction before regional anesthesia in our Caribbean setting.

## Materials and methods

Study design

This study was a prospective randomized controlled trial done at a tertiary-level obstetrics center from November 2019 to June 2020. This study was non-patient blinded. Ethical approval was obtained from the Campus Research Ethics Committee of the University of the West Indies (CEC 1165/06/19) and the North West Regional Health Authority. Written informed consent was obtained from all eligible candidates before enrolment into the study.

The sample size was determined to be 80 patients for a 90% power to determine a > seven-point reduction in the Spielberger State-Trait Anxiety Inventory (STAI) score using a mean (SD) of 41.5 (10.5) with a significance level set at 5%. Sample size determination was based on previous work by Eley et al. [14]. Inclusion criteria included patients who were 18 years and older scheduled for elective cesarean section under regional anesthesia. Exclusion factors included patients who had an emergency cesarean section, non-English speaking, patients with visual or hearing impairment, inability to read and/or write, psychiatric disorder, history of anxiety or depression, and patients scheduled for general anesthesia. Ninety-eight participants were recruited sequentially until we obtained 80 participants who were suitable for data analysis. Participants were randomized using simple randomization, done via the online webpage www.randomization.com.

Patients presenting for elective cesarean section were screened the evening before surgery for eligibility and consent was obtained. Following informed consent, eligible participants entered into their respective groups in accordance with the randomization list.

Study instruments

The STAI questionnaire was used to assess the participant’s anxiety levels. It contains two subscales for measuring the state and trait anxiety, with 20 questions each, scored as follows: 1 point = not at all; 2 = somewhat; 3 = moderately so; and 4 = very much so. For each subscale, the lowest score is 20 points and the highest score is 80 points [15,16].

The visual analog scale for anxiety (VAS-A) was used to measure the state of anxiety. It was presented as a 10 cm line, starting from 0 cm representing no anxiety to 10 cm representing maximum anxiety [17].

The Maternal Satisfaction Scale Score for Cesarean Section (MSSSCS) was used to assess the satisfaction score. It consisted of 22 questions scored on a seven-point Likert score, with a lowest score of 22 and a highest score of 154 [18].

The educational anesthetic video was approximately 10 minutes long and demonstrated the typical journey of a patient from arrival to the operating theatre up until discharge from the recovery room, focusing on the theatre environment, a simple explanation about the neuraxial technique, patient monitoring during surgery, proper position for the neuraxial block, advantages of a neuraxial block, potential complications, and what to expect during the perioperative period, up until discharge from the recovery room. Images of an actual patient having a spinal anesthetic for her cesarean section were used in the video, after obtaining informed consent from the patient and the anesthetist performing the spinal anesthesia.

The video was created after incorporating several still images taken with a smartphone camera into the program Microsoft PowerPoint (Microsoft Corporation, Redmond, WA). Narration and animation were then added to the PowerPoint slides, which were converted to a video using an intrinsic feature of the application, as seen in Video 1.

**Video 1:** Preoperative study video

A non-validated video feedback questionnaire was also completed by the participants. It consisted of five questions scored on a five-point Likert scale to assess the usefulness of the video.

Measurements and procedures

Standard care at our center includes a preoperative anesthetic consultation performed on the morning of surgery, focusing on history, examination, review of investigations, and obtaining informed verbal consent for the planned anesthesia.

To avoid introducing bias, the anesthesiologist performing the preoperative consult was blinded to which group the patient belonged to and the patient was advised to keep this information private to encourage the same amount of information to be provided to all patients as per routine practice. The type of anesthesia was solely determined by the anesthesiologist and the patient.

Baseline Measurements

On the evening before surgery, the baseline anxiety level was assessed using the STAI trait and state questionnaire and the VAS-A score.

Intervention

Participants randomized to the intervention group looked at the educational video on a laptop with disposable headphones. After viewing the video, the VAS-A score was remeasured and a non-validated video feedback questionnaire was completed. This was done the evening before the scheduled surgery.

Preoperative and Postoperative Measurements

The STAI state and VAS-A were measured just before surgery in the holding bay of the maternity operating theater in both groups of patients following their preoperative assessment. Lastly, the MSSCS questionnaire was completed on the first postoperative day between 6 pm to 7 pm.

To avoid any confounding factors, e.g., sleep deprivation, change in appetite, challenges in caring for the newborn, adjusting to a new role, and breastfeeding challenges, the satisfaction score was assessed between 6 pm and 7 pm on the evening of surgery once the patient’s analgesia was well controlled (numerical rating pain scale (NRS) score <3). If the pain score was greater than or equal to 3, analgesia was given. The questionnaire was only completed when the NRS reached <3.

Statistics

Data were analyzed using Statistical Package for the Social Sciences (SPSS) version 27 (IBM Corp., Armonk, NY). Ordinal data were analyzed using the non-parametric tests: Wilcoxon signed rank test and Mann-Whitney U test. The STAI state, VAS-A scores, and MSSSCS were evaluated between the two groups. VAS-A immediately after watching the video was also assessed. Data are presented as median and interquartile range.

## Results

A total of 98 participants were recruited sequentially until a target sample size of 80 participants who were suitable for data analysis was achieved.

**Figure 1:**
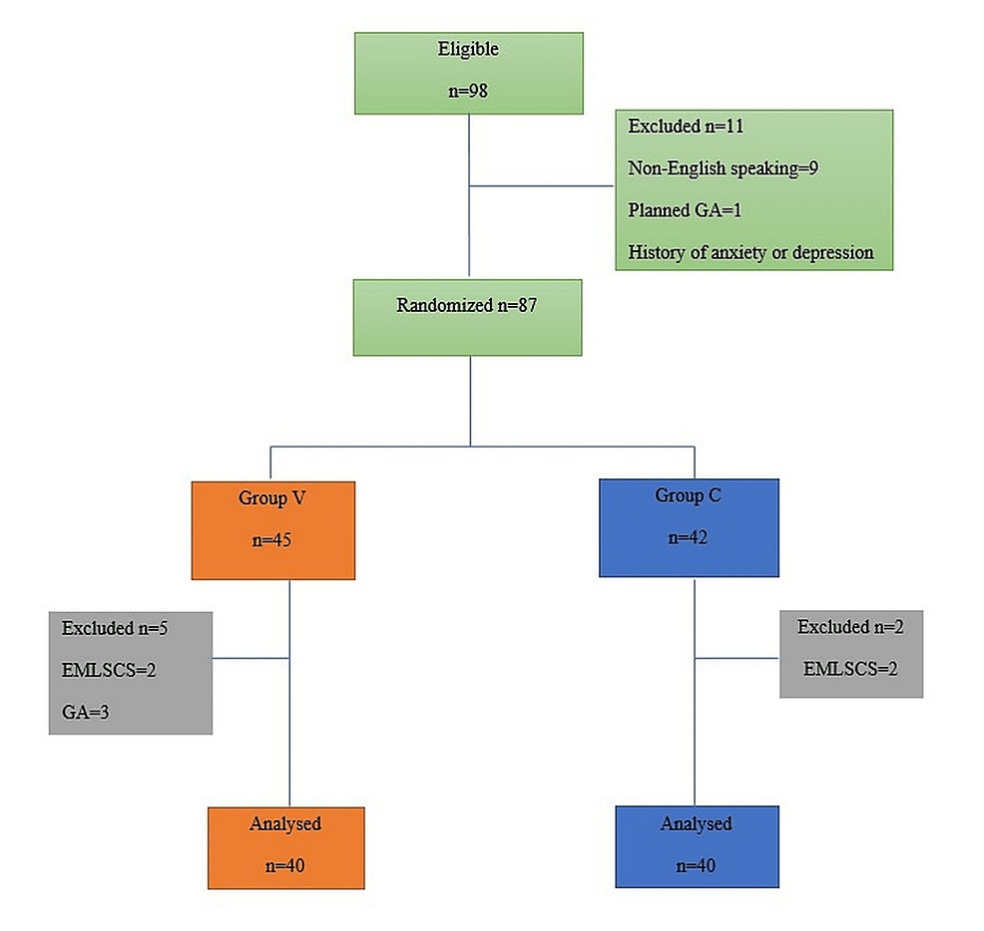
Flowchart of participants EMLSCS, emergency lower-segment cesarean section; GA, general anesthesia.

Eleven participants were excluded after applying the inclusion and exclusion criteria. Of those excluded, nine patients were non-English speaking, one patient was scheduled for planned general anesthesia, one had a history of anxiety and depression, and one patient was hearing impaired. A further seven patients were excluded after entering the study because four participants underwent emergency cesarean section and three had a planned general anesthetic. The flow of participants that were enrolled is shown in Figure 1.

Baseline data

Both the control group and the intervention group were closely matched at baseline (Table 1).

**Table 1:** Baseline data between groups Age is displayed as mean and standard deviation (SD). Parity, STAI trait, and ASA classification are displayed as median (interquartile range (IQR)). STAI, Spielberger State-Trait Anxiety Inventory; ASA, American Society of Anesthesiologists; LSCS, lower segment cesarean section; CNB, central neuraxial block.

Variable	Control group	Intervention group	P-value
Age, SD	30.9, 5.98	32, 6.14	0.409
Parity (IQR)	1 (1-2)	2 (1-3)	0.297
STAI trait (IQR)	45.5 (41-50)	46.9 (42.2-50.0)	0.721
ASA classification (IQR)	2 (2-2)	2 (2-2)	0.155
Partner’s support (n, %)	40, 100	36, 90	0.116
Family support (n, %)	38, 95	36, 90	0.675
Employed (n, %)	27, 67.5	26, 65.0	0.813
Education up to primary school (n, %)	3, 7.5	5, 12.5	0.712
Education up to secondary school (n, %)	18, 45	21, 52.5	0.502
Education up to tertiary school (n, %)	19, 47.5	14, 35	0.256
Married (n, %)	36, 90	32, 80	0.210
Previous LSCS (n, %)	27, 67.5	21, 52.5	0.17
Previous surgery (n, %)	33, 82.5	30, 75	0.412
Previous CNB (n, %)	25, 62.5	23, 57.5	0.648
No previous CNB (n, %)	15, 37.5	17, 42.5	0.648
Fetal anomaly (n, %)	1, 2.5	0, 0	0.314

Baseline anxiety was elevated in both groups with no statistical difference. The median STAI trait score was 45.5 (41-50) vs. 47.5 (42.2-50) (p = 0.721) in the control group vs. intervention group, respectively. A history of a previous cesarean section was comparable in both groups (control group vs. intervention: 67.5% vs. 52.5%; p = 0.17), as depicted in Table 1. A similar number of patients had no prior central neuraxial block in the control group vs. intervention group (37.5% vs. 42.5%; p = 0.648). An extremely low incidence of fetal anomaly was seen in both groups (2.5% vs. 0%; p = 0.314). Intraoperative data are shown in Table 2.

**Table 2:** Intraoperative variables between groups The number of attempts at CNB and the NRS pain score in the recovery room are displayed as median (interquartile range (IQR)). CNB, central neuraxial block; NRS, numerical rating scale.

Intraoperative variables (n, %)	Control group (n = 40)	Intervention group (n = 40)	P-value
Spinal (n, %)	24, 60	25, 62.5	0.818
Combined spinal epidural (n, %)	15, 37.5	14, 35	0.816
Epidural (n, %)	1, 2.5	0, 0	1
Block failure (n, %)	1, 2.5	0, 0	0.314
Conversion to general anesthetic (n, %)	1, 2.5	2, 5.0	0.560
Intraoperative supplemental analgesic required (n, %)	10, 25	7, 17.5	0.586
Number of attempts at CNB (IQR)	2 (1-3)	2 (1-3)	0.609
NRS pain score in the recovery room (IQR)	0 (0-0)	0 (0-0)	0.980

There was a low and non-statistically significant difference in the incidence of block failure in the control group (n = 1) vs. intervention group (n = 0; p = 0.314), and a negligible rate of conversion to general anesthesia (n = 1 vs. n = 2; p = 0.560). Likewise, the need for supplemental analgesia was equivalent in both groups (n = 10 vs. n = 7; p = 0.586).

Outcomes

As can be seen in Table 3, there was a statistically significant reduction in the 10-point VAS-A score as observed in the intervention group vs. the control group (6 vs. 4.6, p = 0.018).

**Table 3:** Outcome measures STAI, Spielberger State-Trait Anxiety Inventory; VAS, visual analog scale; MSSSCS, Maternal Satisfaction Scale Score for Cesarean Section.

Variable, median (IQR)	Control	Intervention	P-value
STAI state	46.5 (41.25-50.00)	44.0 (41-50)	0.988
VAS anxiety score before surgery	6 (4-8)	4.6 (1-7)	0.018
MSSSCS	104.5 (92.5-120)	113 (103.5-126.5)	0.015
VAS anxiety score before* and after** looking at the video		*5 (1-6) and **4 (1-6)	0.323

There was also a statistically significant increase in maternal satisfaction in the intervention vs. the control group (113 vs. 104.5; p = 0.015). State anxiety measured by the STAI state score was not significantly different between intervention and control groups (44 vs. 46; p = 0.99). VAS-A before and after looking at the video was not statistically significant (5 vs. 4; p = 0.323).

Video feedback

The results from the non-validated feedback questionnaire about the video are shown in Table 4.

**Table 4:** Video questionnaire feedback Rating scale: 1 - strongly disagree; 2 - disagree; 3 - neutral; 4 - agree; 5 - strongly agree.

Video feedback questions	Median (IQR)
The video was informative.	5 (5-5)
The content was sufficient.	5 (4-5)
The video was easy to understand.	5 (5-5)
The video reduced my fear or worry about anesthesia.	5 (4-5)
Overall, I found the video useful and would recommend its continued use.	5 (5-5)

A median score of 5 (“strongly agree”) was reported by participants to the five questions used to assess the video’s content.

## Discussion

A high level of anxiety was detected among patients having an elective cesarean section at our center, with a median STAI state score of more than 40 in both groups. This is in keeping with the international data available that show a high prevalence of preoperative anxiety in up to 60-80% of patients [19].

The evidence showing an anxiety reduction following preoperative education is variable and may be due to differences in methodology, the information provided, the patient’s learning capability, and subsequent understanding [19,20]. Therefore, the methods used must be specifically suited to the target patient population. Of note, only 40% of the patients studied had a tertiary-level education.

In this study, the use of the preoperative video did result in a statistically significant reduction of maternal anxiety using the VAS-A; however, this was not so when measured with the STAI state questionnaire. There are many validated tests available to measure a patient’s anxiety level, with the STAI and VAS-A being commonly used and well-validated to assess preoperative anxiety [17,21]. Many studies have demonstrated that the STAI questionnaire has good content validity and is a reliable test for assessing patient anxiety, including one study done in Jamaica, within the Caribbean [15,16,21,22]. However, it was observed from this study that certain questions were unclear to some patients, such as "I am a steady person" and "I feel inadequate," leading to differing interpretations that may affect the results obtained. It is possible that further validation and modification may be needed in a Caribbean setting. On the other hand, the single item VAS-A is a simple and quick test; patients were asked a single question and they placed an "X" at the appropriate point on the line. Consequently, it may be less likely to be affected by differences in interpretation.

In this study, the educational anesthetic video provided patients with information at an earlier time, on the evening before surgery rather than on the morning of surgery. This may also contribute to the reduction in anxiety observed as studies revealed that earlier preoperative education reduces anxiety [23]. Performing the preoperative consultation on the morning of surgery leaves limited time for patient assessment, providing information, and addressing patients' questions and concerns.

A statistically significant increase in maternal satisfaction was also seen in patients who looked at the educational video vs. those in the control group. Although maternal satisfaction is a complex issue, several studies have also demonstrated an increase in maternal satisfaction following the use of an educational video [11,13,24,25]. Hobson et al. [26] also demonstrated an inverse relationship between preoperative anxiety and maternal satisfaction in patients undergoing cesarean section.

Video is an advantageous format of preoperative education that incorporates both audio and visual features and is an effective way of transferring information. It may be more beneficial than using written and verbal instruction only, especially in patients with reduced learning capabilities and difficulty reading and understanding. Furthermore, it is a convenient way to deliver information as it can be shown to patients at their convenience without necessarily having the anesthesiologist present.

Almost all patients strongly agreed that the video used was informative, had sufficient content, was easy to understand, reduced their fear about anesthesia, and would recommend its continued use. Overall, it was well received and appreciated by the patients. Additionally, the video was created using only a smartphone and a single computer application thus making it easily reproducible in other centers. Although adequate patient information content may be found in a variety of sources, specifically designed content can be easily made, as in our case, and may be more culturally relevant to the specific patient population.

Limitations

In this study, there was a considerable number of patients who had previous central neuraxial blocks and cesarean sections in both the control and intervention groups. Previous experiences with surgery and regional anesthesia may have affected the participant's perioperative anxiety apart from the intervention used. However, despite this, both intervention and control groups were equally matched with no statistically significant difference in the rate of cesarean sections and regional anesthesia between groups.

The education video used in our study presented new information to the patients before surgery. It is possible that some patients may not have understood what was being presented to them in the video and so decreasing the efficacy of the intervention. We did not directly assess the patient's understanding following the video; however, the majority of participants "strongly agreed" that the video was easy to understand and useful (Table 4). When designing an educational video, dedicated feedback from a cohort of patients would be useful to determine if the intervention is appropriate to meet its intended goals.

## Conclusions

Cesarean section under regional anesthesia in our cohort of patients is associated with significant preoperative anxiety. The addition of a short preoperative anesthetic video was shown to reduce preoperative anxiety as measured by the VAS-A scale. No significant reduction in anxiety was noted when using the STAI questionnaire. Maternal satisfaction assessed by the MSSSCS was also significantly reduced in those viewing the video. This video can be easily created using a smartphone camera and a single computer application. This video format is versatile and adaptable to a wide variety of cultural contexts where it can pre-empt and supplement the information given by anesthesiologists.
